# Immunogenicity of influenza vaccines administered to pregnant women in randomized clinical trials in Mali and South Africa

**DOI:** 10.1016/j.vaccine.2020.07.020

**Published:** 2020-09-22

**Authors:** Avnika B. Amin, Marta C. Nunes, Milagritos D. Tapia, Shabir A. Madhi, Clare L. Cutland, Niteen Wairagkar, Saad B. Omer, for BMGF Supported Maternal Influenza Immunization Trials Investigators Group

**Affiliations:** a Department of Epidemiology, Emory University Rollins School of Public Health, Atlanta, USA; b Medical Research Council: Respiratory and Meningeal Pathogens Research Unit, University of the Witwatersrand, Johannesburg, South Africa; c Department of Science and Technology—National Research Foundation, Vaccine-Preventable Diseases, Johannesburg, South Africa; d Centre pour le Développement des Vaccins, Bamako, Mali; e University of Maryland School of Medicine, Center for Vaccine Development, Baltimore, USA; f Bill & Melinda Gates Foundation, Seattle, USA; g Hubert Department of Global Health, Emory University Rollins School of Public Health, Atlanta, USA; h Emory Vaccine Center, Atlanta, USA; i Department of Pediatrics, Emory University School of Medicine, Atlanta, USA

**Keywords:** Influenza, Influenza vaccine, Maternal vaccination, Immunogenicity, Transplacental antibody transfer

## Abstract

**Background:**

A key consideration for expanding recommendations for influenza vaccination is a robust assessment of immunogenicity and efficiency of transplacental antibody transfer after maternal vaccination.

**Methods:**

We pooled data from two trials of maternal influenza vaccination to analyze vaccine immunogenicity with more power than either trial had alone. We compared hemagglutination-inhibition (HAI) titers and titer factor change for women and their infants between trial arms using t-tests; maternal and infant putative seroprotective titers (HAI ≥ 1:40) within each trial arm and maternal seroconversion between trial arms using exact tests; and transplacental antibody transfer between trial arms using t-tests. We used marginal linear models and generalized estimating equations to examine the impact of time between maternal vaccination and delivery on transplacental antibody transfer, infant titers, and infant seroprotection.

**Results:**

For all vaccine components (A/H1N1, A/H3N2, and Type B), >80% of vaccinated women had seroprotective titers, >60% of them seroconverted, and >50% of their infants were born with seroprotective titers. These immunogenicity outcomes occurred more often in vaccine recipients and their infants than in controls. No difference in efficiency of transplacental antibody transfer was observed between vaccine recipients and controls.

**Conclusions:**

Our results provide robust support for further expansion of maternal influenza vaccination recommendations.

Clinical Trials Registration: NCT01430689 and NCT01306669.

## 1 Background

Administration of inactivated influenza vaccines (IIV) has been evaluated as a strategy to prevent influenza-associated adverse outcomes among pregnant women and their young infants [1], [2], [3]. In clinical trials, maternal IIV was associated with prevention of clinical pneumonia and PCR- and rapid-test confirmed influenza among infants younger than six months of age. Similarly, maternal IIV is associated with prevention of laboratory-confirmed influenza among pregnant women [2], [4].

While several countries have recommendations for routinely vaccinating pregnant women against influenza, a substantial proportion of the global cohort of pregnant women is not covered by these recommendations [5]. Specifically, most countries in Sub-Saharan Africa and South Asia, including Mali and South Africa, do not have a recommendation to vaccinate pregnant women against influenza.

One consideration for expanding recommendations for maternal IIV to a variety of settings is a robust assessment of immunogenicity and efficiency of transplacental antibody transfer after IIV in pregnancy. Establishment of IIV seroprotection in healthy populations is already nuanced, as there is no clear absolute correlate of protection [6]. Hemagglutination-inhibition antibody (HAI) titers of ≥1:40 correspond to approximately 50% protection in younger adults [7], although whether this threshold’s meaning applies to high-risk populations like pregnant women is unclear. There are few studies of maternal IIV in low- and middle-income countries, and current evidence on immunogenicity is nuanced. For example, in a randomized controlled trial of the trivalent inactivated influenza vaccine (IIV3) administered to Bangladeshi pregnant women in the third trimester of pregnancy, the response to vaccination was differential by antigen [8]. However, the efficiency of IgG transfer from mother to infant was consistent across the three antigens [8].

Two recent trials of maternal IIV3 conducted in Mali and South Africa provide an opportunity to evaluate immunogenicity in a variety of low-income settings. The two trials ended up using vaccines with identical strains for at least a portion of the trial period, providing a unique opportunity to pool data and evaluate overall and strain-specific immunogenicity-related research questions [9], [10]. Moreover, the pooling of mother-infant pairs provides an opportunity to perform immunogenicity analyses that had lower statistical power in individual analyses of these trials [11].

In this study, we evaluated the pooled immunogenicity of maternal IIV using data from two randomized controlled trials conducted in Sub-Saharan Africa. The primary objective of this analysis was to assess the relationship between maternal IIV3 receipt and (1) maternal and infant HAI antibody titers, (2) maternal and infant factor changes in HAI titers, (3) maternal and infant putative seroprotection, (4) maternal seroconversion, and (5) transplacental antibody transfer. The secondary objective of this analysis was to explore the relationship between time from IIV3 receipt to delivery and (1) infant HAI titers at birth, (2) infant putative seroprotection at birth, and (3) transplacental transfer for IIV3 recipients and their infants.

## 2 Methods

### 2.1 Study population

Study participants were women enrolled in IIV3 trials in South Africa (NCT01306669) and Mali (NCT01430689). Previous publications have described both trials [7], [8], [10]. Written informed consent was obtained from all participants, and the protocols and other materials were approved by independent ethics committees and institutional review boards.

**Fig. 1:**
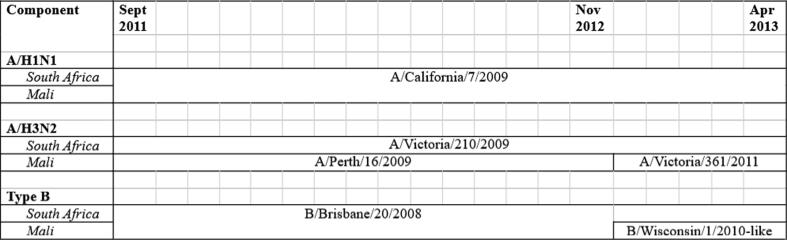
**Timing of influenza strains used in the vaccines (Vaxigrip, Sanofi Pasteur) during each trial.** The same A/H1N1 strain was used for the duration of both trials. Three different A/H3N2 strains and two different Type B strains were used.

Briefly, women accessing prenatal care were screened and enrolled in Bamako, Mali (gestational age ≥28 weeks) and Soweto, South Africa (gestational age 20–36 weeks). They were randomized to receive either IIV3 containing A/H1N1, A/H3N2, Type B components (Vaxigrip, Sanofi Pasteur) or a control (quadrivalent meningococcal vaccine in Mali, saline injection in South Africa). The same A/H1N1 strain (California/7/2009) was used in the vaccine for the entirety of both trials, while A/H3N2 strains (Victoria/210/2009, Perth/16/2009, and Victoria/361/2011) and B lineages (Brisbane/20/2008 and Wisconsin/1/2010-like) used in the vaccine formulations varied. Fig. 1 details when each strain was used during the trials.

Women and their infants were followed until six months postpartum. Active surveillance for influenza was conducted by weekly contact with participants for all of follow-up. Those reporting influenza-like illness were tested for subtype-specific influenza infection. Common timepoints for blood sample collection occurred at baseline (women only), one month post-vaccination (women only), delivery (±7days; women and infants), and six months postpartum (infants only).

### 2.2 Statistical analyses

Our analyses focus on immunogenicity outcomes appropriate for three groups of study participants: women only, mother-infant pairs, and infants only. We analyzed five outcomes for each vaccine component: hemagglutination-inhibition (HAI) antibody titers, factor change in HAI between visits, proportion of participants with putative seroprotective titers, proportion of seroconverted women, and ratio of infant:mother antibody at delivery (transplacental antibody transfer). We define an HAI titer of at least 1:40 as a relative putative measure of seroprotection and at least a fourfold increase in HAI between visits as seroconversion. Pooling of trial data combined the three H3N2 strains for A/H3N2 component analyses and the two B lineages for Type B component analyses (Fig. 1). Analyses were conducted in SAS 9.4 (Cary, NC).

For women-only analyses, we included women who had titers recorded for both baseline and one-month post-vaccination visits. Women were excluded from all analyses if the one-month visit occurred <28 or >35 days after vaccination, or from component-specific analyses if they tested positive for the influenza virus component prior to the one-month visit. For example, a woman positive for H1N1 prior to the one-month visit would be excluded from A/H1N1-specific analysis, but not from A/H3N2 or Type B component analyses. Titers were compared at baseline and one month after IIV3 or control receipt. We performed two-sided, two-sample t-tests to compare geometric mean HAI titers at each visit and the factor change in titer between IIV3 and control groups, using a log-normal distribution for titers and factor change. We used exact tests to compare the proportion of women putatively seroprotected for each component within IIV3 and control groups at each visit, and to compare the proportion who seroconverted between IIV3 and control groups.

For maternal-infant pair analyses, we included pairs if both the mother and the infant had titers recorded within 7 days of delivery. Pairs were excluded from component-specific analyses if the mother tested positive for the component prior to delivery. We performed two-sided, two-sample t-tests to compare the geometric means of the ratio of mother-infant antibody transfer, using a log-normal distribution for the ratio (infant:mother).

For infant-only analyses, we included infants who had titers recorded for both delivery and six-month postpartum visits. Infants were excluded from all analyses if the delivery visit occurred >7 days after birth or if the six-month visit was <168 or >190 days after birth, and from component-specific analyses if the mother tested positive for the influenza virus component prior to delivery or if they tested positive for an influenza virus component between the delivery and six-month visits. For example, an infant whose mother was positive for A/H1N1 infection while pregnant would be excluded from A/H1N1-specific analysis, but not from A/H3N2 or Type B component analyses. An infant who was positive for A/H1N1 infection would be similarly excluded from A/H1N1, but not A/H3N2 or Type B, analysis. Infant titers were compared at delivery and 6 months of age. We performed two-sided, two-sample t-tests to compare the geometric means of the component-specific HAI titers at each visit and the factor change in titer between IIV3 and control groups, using a log-normal distribution for titers and factor change. We used exact tests to compare the proportion of putatively seroprotected infants for each component within IIV3 and control groups at each visit.

We also wanted to examine the impact of the length of time between maternal vaccination and infant birth on immunogenicity outcomes, so we conducted a subanalysis on vaccinated women and their infants only. We used marginal linear models to examine length of time’s effect on transplacental antibody transfer and infant HAI titers at delivery. We used generalized estimating equations to model the effects of IIV3 and length of time between vaccination and birth on infant putative seroprotection at delivery. All models used study site as the clustering variable and a compound symmetric correlation structure. We also evaluated the minimum time needed for prepartum maternal vaccination to see no additional meaningful increase in probability of putative infant seroprotection at birth. We dichotomized weeks between vaccination and birth iteratively between ±1 and 8 weeks and calculated risk ratios comparing putative infant seroprotection at birth after and before the cutoff.

### 2.3 Sensitivity analyses

We conducted three sets of sensitivity analyses for some of the immunogenicity outcomes from the main analyses: maternal putative seroprotection before and after vaccination, maternal seroconversion, transplacental transfer (second analysis only), and infant putative seroprotection at delivery. The first sensitivity analysis assessed the impact of including those who tested positive for IIV3 components in the analyses. Women and infants excluded from the main analyses of maternal putative seroprotection before and after vaccination, maternal seroconversion, and infant putative seroprotection at delivery analyses due to component-specific influenza infection were added to participants included in the main analyses. The statistical tests used in the main analyses were used for their respective outcomes in the influenza-positive sensitivity analyses.

The second sensitivity analysis divided the A/H3N2 and Type B component analyses based on exact strain. We did not conduct strain-specific analyses for A/H1N1 because the same strain was used in all vaccine formulations. We decided against statistical testing, as some influenza strains were received by small numbers of participants. This sensitivity analysis examined all the outcomes in the first sensitivity analysis, as well as the transplacental transfer outcome.

Finally, the third sensitivity analysis assessed the influence of pre-existing HAI titers on maternal response to vaccination. We analyzed the maternal HAI titers at the one month post-vaccination visit and the maternal HAI titer factor change outcomes, stratifying by whether a woman had existing HAI titers at baseline (defined as a titer >1:10).

## 3 Results

We analyzed 404, 383, and 384 women for the A/H1N1, A/H3N2, and Type B component women-only analyses, respectively; 367, 328, and 333 pairs for the A/H1N1, A/H3N2, and Type B component maternal-infant pair analyses, respectively; and 239, 206, and 216 infants for the A/H1N1, A/H3N2, and Type B component analyses, respectively, in our main analyses. The majority of participants − 70% and 80% of participants in women- and infant-only analyses, and approximately 60% of maternal-infant pairs - included in each set of analyses came from the South Africa site (Supplementary Table 1). The median age of women included in women-only analyses was comparable between the South Africa and Mali sites (South Africa median: 26, interquartile range: 23–31; Mali median: 22, interquartile range: 19–28) and between the trial arms at each site [9], [10].

### 3.1 Maternal analyses

**Fig. 2:**
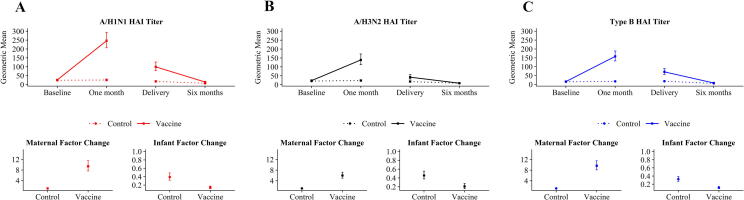
**Geometric mean HAI titers and factor change between measurements by vaccine group.** Panels (A), (B), and (C) display the results for the A/H1N1, A/H3N2, and Type B components of the influenza vaccine, respectively. I bars indicate 95% confidence intervals. ‘Control’ indicates women who received either meningococcal vaccine or a saline injection, or their infants. ‘Vaccine’ indicates women who received trivalent inactivated influenza vaccine, or their infants. Two-sided, two-sample t-tests were used to compare the HAI titers and factor change for each component between control and IIV3 recipient groups, with a log-normal distribution specified for the titers and factor change. Abbreviations: HAI, hemagglutination-inhibition; IIV3, trivalent inactivated influenza vaccine.

Control and IIV3 recipient titers were similar at baseline for all three IIV3 components, but IIV3 recipient titers were much higher post-vaccination than control titers (Fig. 2, Supplementary Table 2). IIV3 recipient post-vaccination titers increased from baseline by a factor of 9.4, 6.0, and 9.6 for A/H1N1, A/H3N2, and Type B components, respectively. 93%, 83%, and 91% of IIV3 recipients had putatively seroprotective titers for A/H1N1, A/H3N2, and Type B components, respectively, at the one-month post-vaccination visit. (Fig. 2, Supplementary Table 3). 77.0%, 64.8%, and 84.5% of IIV3 recipients seroconverted between baseline and post-vaccination for A/H1N1, A/H3N2, and Type B components respectively, compared to 6.2%, 3.2% and 3.7% of control women (Fig. 2, Supplementary Table 3).

### 3.2 Mother-infant pair analyses

**Fig. 3:**
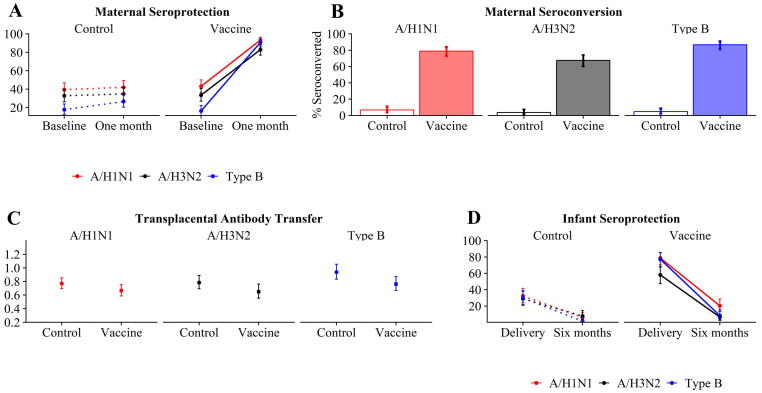
**Seroprotection, seroconversion, and transplacental antibody transfer by vaccine component.** Panels (A) and (D) display the proportion of women and infants, respectively, with a seroprotective titer of at least 1:40. Panel (B) shows the proportion of women who seroconverted (at least a fourfold increase in titers) between baseline and one month post-vaccination visits. Panel (C) shows the ratio of infant:mother antibodies at birth. Two-sided exact tests were used to compare the proportion seroprotected against each vaccine component within control and vaccine recipient groups, and the proportion who seroconverted for each component between groups. Two-sided, two-sample t-tests were used to compare the transplacental transfer of hemagglutination-inhibition antibodies for each vaccine component between control and IIV3 recipient groups, with a log-normal distribution specified for transfer ratio.

The efficiency of antibody transfer did not meaningfully differ between IIV3 recipient and control mother-infant pairs for any of the three influenza vaccine components (Fig. 3, Supplementary Table 4). Longer time between maternal IIV3 receipt and birth was associated with higher transplacental antibody transfer (Table 1).

**Table 1:** Relationship of time from prepartum vaccination to infant’s birth with transplacental antibody transfer and infant HAI titers at delivery. Both model results were generated from marginal linear models using a compound-symmetric correlation matrix and of form *Outcome_ij_ = β_0_ + β_1_(weeks_postvax_ij_)*, where *i* represents the cluster variable (trial site) and *j* represents the within-cluster index (participant). *Weeks_postvax* was treated as a continuous variable. Bold coefficients represent a statistically significant result.

Outcome	A/H1N1		A/H3N2		Type B	
	β	SE	β	SE	β	SE
Transplacental antibody transfer^1^	**0.05**	0.01	**0.06**	0.02	**0.03**	0.01
Infant HAI titers at delivery^2^	0.03	0.02	0.03	0.03	0.03	0.02

Abbreviations: SE, standard error; HAI, hemagglutination-inhibition antibody.

1 The specific outcome, ratio of infant:maternal antibody at delivery, was specified to have a log-normal distribution with an identity link. Example interpretation of the beta coefficient in the A/H1N1 model: Each additional week between maternal vaccination and birth was associated with an average 0.05 increase in the ratio of infant:maternal antibody.

2 The specific outcome, HAI titer, was specified to have a log-normal distribution with an identity link. Example interpretation of the beta coefficient in the A/H1N1 model: Each additional week between maternal vaccination and birth was associated with a 0.03 average increase in infant HAI titers.

### 3.3 Infant analyses

Infants of IIV3 recipients had higher titers at delivery than those of controls (Fig. 2, Supplementary Table 2). However, infants in the IIV3 and control groups had similar titer levels by six months of age. 82%, 60%, and 81% of IIV3 infants had putatively seroprotective titers for A/H1N1, A/H3N2, and Type B, respectively, at delivery (Fig. 3, Supplementary Table 4). Longer time between maternal IIV3 receipt and birth was associated with higher HAI titers and likelihood of putative seroprotection at birth (Table 2, Table 3). More specifically, maternal vaccination at least 2–4 weeks prepartum appeared to be the minimum time needed to maximize likelihood of putative infant seroprotection at birth was apparent (Table 3).

**Table 2:** Relationship between time from prepartum vaccination to infant’s birth and infant seroprotection (HAI titer ≥ 1:40) at delivery. Model results were generated from generalized estimating equations using a compound-symmetric correlation matrix and of form *Seroprotection_ij_ = β_0_ + β_1_(weeks_postvax_ij_)*, where *i* represents the cluster variable (trial site) and *j* represents the within-cluster index (participant). The outcome was specified to have a binomial distribution with a logit link. *Weeks_postvax* was treated as a continuous variable.

Covariate	A/H1N1		A/H3N2		Type B	
	OR	95% CI	OR	95% CI	OR	95% CI
Weeks between maternal vaccination and birth	1.07	(0.99, 1.16)	1.06	(0.97, 1.16)	1.04	(0.97, 1.12)

Example interpretation of the OR in the A/H1N1 model: Each additional week between maternal vaccination and birth was associated with a 7% increase in the odds of infant seroprotection at birth for infants whose mothers received IIV3.

Abbreviations: HAI, hemagglutination-inhibition; OR, odds ratio; CI, confidence interval; IIV3, trivalent inactivated influenza vaccine.

**Table 3:** Evaluation of minimum time from prepartum vaccination to infant’s birth to maximize likelihood of infant seroprotection (HAI titer ≥ 1:40) at delivery.

Comparison^a^	A/H1N1		A/H3N2		Type B	
	RR	95% CI	RR	95% CI	RR	95% CI
>1 vs ≤1 week	2.23	(1.02, 4.89)	2.31	(0.89, 6.00)	8.30	(1.28, 53.91)
>2 vs ≤2 weeks	1.66	(1.12, 2.45)	1.26	(0.85, 1.88)	2.55	(1.37, 4.76)
>3 vs ≤3 weeks	1.45	(1.08, 1.95)	1.13	(0.82, 1.55)	1.95	(1.24, 3.04)
>4 vs ≤4 weeks	1.28	(1.02, 1.61)	1.12	(0.85, 1.50)	1.55	(1.11, 2.15)
>5 vs ≤5 weeks	1.18	(0.98, 1.42)	1.02	(0.80, 1.31)	1.42	(1.08, 1.89)
>6 vs ≤6 weeks	1.11	(0.94, 1.30)	0.90	(0.73, 1.11)	1.21	(0.97, 1.50)
>7 vs ≤7 weeks	1.08	(0.93, 1.25)	0.96	(0.78, 1.19)	1.16	(0.95, 1.42)
>8 vs ≤8 weeks	1.07	(0.92, 1.23)	0.89	(0.72, 1.10)	1.19	(0.99, 1.45)

An example interpretation of the RR for the >1 vs ≤1 week comparison for A/H1N1: The probability of an infant being seroprotected at delivery when born more than one week after mother’s IIV3 receipt is 2.23 times higher than if the mother had been vaccinated no more than one week before delivery.

Abbreviations: HAI, hemagglutination-inhibition; RR, risk ratio; CI, confidence interval.

a Each comparison row refers to dichotomization of the weeks between maternal vaccination and delivery.

### 3.4 Sensitivity analyses

Inclusion of influenza-positive women, maternal-infant pairs, and infants did not meaningfully shift the results from the main analyses for putative maternal seroprotection, maternal seroconversion, or putative infant seroprotection at delivery (Supplementary Tables 5 and 6). Strain-specific analyses yielded results similar to the main analyses for putative maternal seroprotection and seroconversion, transplacental transfer, and putative infant seroprotection at delivery (Supplementary Tables 7 and 8). Pre-existing immunity stratified analyses indicated that regardless of pre-existing immunity, women who received IIV3 experienced an increase in titers greater than that observed among women who received the control (Supplementary Table 9). The titer increase was more pronounced for immunologically-naïve women.

## 4 Discussion

IIV3 was immunogenic for all three vaccine components, with strongest responses to the A/H1N1 component. These results are consistent with the previously-published immunogenicity analyses of trial data from South Africa [9], [12]. The weaker response to A/H3N2 and Type B components was likely not due to the use of different vaccine strains for these two components when data were pooled, as putative seroprotection and seroconversion by strain reflect the general pattern in the main analyses. Although infant antibody decay appeared larger for infants of IIV3 recipients, these infants started at a much higher titer than infants of controls. Overall, our results for maternal and infant immunogenicity support the work on the individual trials previously published [9], [10], [12].

Few studies have examined immunogenicity of maternal influenza vaccination in relation to the timing of vaccine administration. While one randomized trial suggested that vaccination earlier in pregnancy resulted in lower infant titers at delivery, two other trials did not find any associations [13], [14], [15]. Previous analysis of the South Africa trial data indicated that more time between maternal vaccination and delivery had a modest, positive impact on transplacental antibody transfer and infant titers at delivery [11]. Our similar results lend further support for earlier prepartum vaccination within the gestational age range eligible for trial enrollment.

One limitation is the number of observations that were excluded from pooled analyses due to late or missing follow-up. Although there were nearly 600 maternal records at baseline, nearly one-third of them were excluded due to either missing post-vaccination data or titer data collected outside of the 7-day window for the one-month post-vaccination visit. These women lost to follow-up may be substantially different in their immunogenic responses than the women included with follow-up data, potentially biasing our results. We did not vary acceptable windows for blood sample collection given the sensitivity analyses already conducted. We are also unable to draw conclusions about vaccine immunogenicity for HIV-infected individuals or for vaccine administration earlier in pregnancy, as only HIV-uninfected women at 20 weeks gestation or later were included in this analysis. The majority of the data came from the South Africa trial, which yielded results similar to those from only South Africa.

Information on factors that could potentially affect vaccine immunogenicity, like nutritional status and influenza illness and vaccination history, were unavailable. As the distribution of maternal age was similar between sites and between trial arms, suggesting successful randomization of this factor, concerns about potential differences in the distribution of unmeasured factors between trial sites is mitigated. Although we defined putative seroprotection at HAI titers ≥1:40, this threshold should be further explored among pregnant women and infants as it was established in studies of healthy young adults and may not apply to these more vulnerable populations.

In conclusion, our results demonstrate that influenza vaccination of pregnant women was immunogenic for both mothers and infants. Infants of vaccinated women were more likely to be born with putatively seroprotective titers, and prior pooled analyses of these trials indicate that these infants experienced lower influenza-related morbidity and mortality [16]. Women in low- and middle-income countries often present for prenatal care later in pregnancy, which may reduce the benefits they and their infants receive from maternal vaccination. This concern is mitigated by the minimum of only two to four weeks needed between maternal vaccination and birth for substantial putative infant seroprotection when delivered. Our results support the pursuit of maternal vaccination as an infant influenza prevention strategy in developing countries.

## Potential conflicts of interest

A.B.A., M.D.T., S.A.M., C.L.C., N.W., and S.B.O. report no potential conflicts of interest. M.C.N. reports personal fees from Sanofi Pasteur and Pfizer and grants from MedImmune outside the submitted work.

## Financial support

The trials in Mali and South Africa were supported by the Bill & Melinda Gates Foundation (grant numbers OPP1002744 and OPP1002747, respectively). The pooled analysis received funding from the Bill & Melinda Gates Foundation (contract number 24090).

## Declaration of Competing Interest

The authors declare that they have no known competing financial interests or personal relationships that could have appeared to influence the work reported in this paper.
