# Supplementary material for: Immunogenicity of influenza vaccines administered to pregnant women in randomized clinical trials in Mali and South Africa
Source: Vaccine. 2020 Sep 22;38(41):6478–83. doi: 10.1016/j.vaccine.2020.07.020 (PMC7505225; doi:10.1016/j.vaccine.2020.07.020)
Supplement: Supplementary data 1 [file mmc1.docx]

**Supplementary Material**

| **A** | **B** | **C** |
| --- | --- | --- |
| **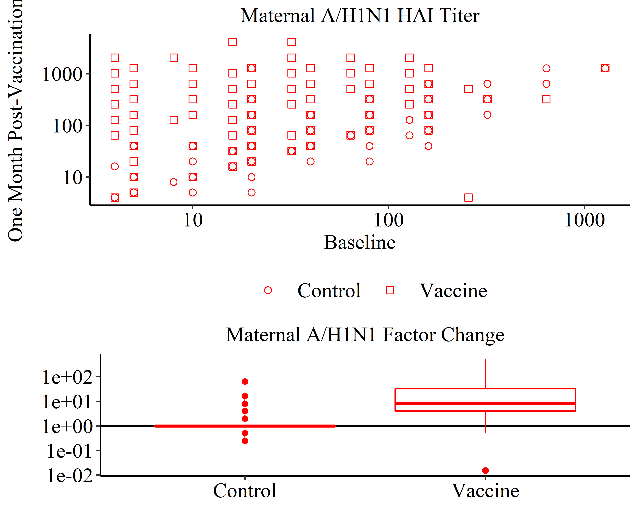** | **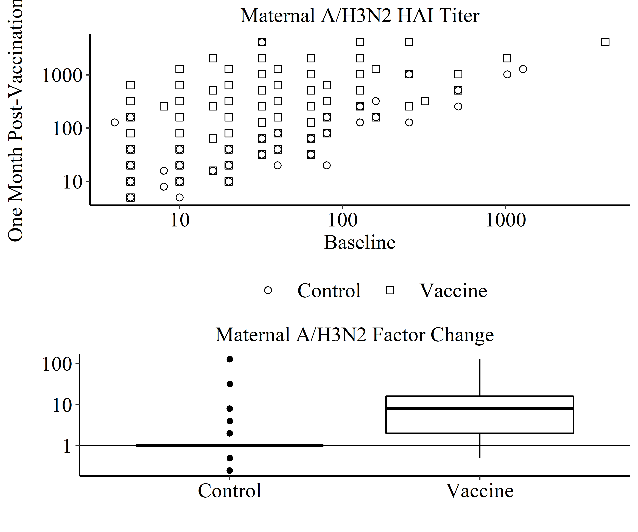** | **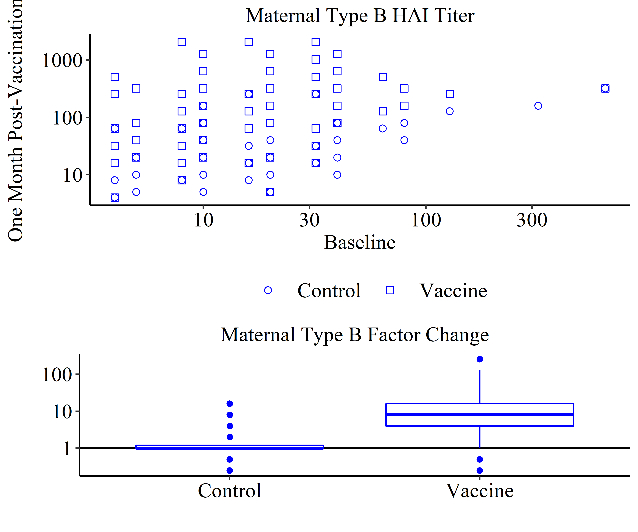** |

**Supplementary Figure 1.** **Distribution of HAI titers and factor change for women.** Panels (A), (B), and (C) display the results for the A/H1N1, A/H3N2, and Type B components of the influenza vaccine, respectively. ‘Control’ indicates women who received either meningococcal vaccine or a saline injection. ‘Vaccine’ indicates women who received trivalent inactivated influenza vaccine. Abbreviations: HAI, hemagglutination-inhibition

**Supplementary Table 1.** Number of participants from each study site included in main analyses.

|  | **A/H1N1** | **A/H3N2** | **Type B** |
| --- | --- | --- | --- |
|  | *N (%)* | | |
| Women^a^ |  |  |  |
| *South Africa* | 290 (71.8) | 287 (74.2) | 289 (74.5) |
| *Mali* | 114 (28.2) | 100 (25.8) | 99 (25.5) |
| *Total* | 404 | 386 | 388 |
| Infants^b^ |  |  |  |
| *South Africa* | 170 (70.8) | 164 (79.2) | 164 (75.2) |
| *Mali* | 70 (29.2) | 43 (20.8) | 54 (24.8) |
| *Total* | 240 | 207 | 218 |
| Maternal-Infant Pairs^c^ |  |  |  |
| *South Africa* | 211 (57.2) | 209 (63.3) | 211 (62.8) |
| *Mali* | 158 (42.8) | 121 (36.7) | 125 (37.2) |
| *Total* | 369 | 330 | 336 |
| ^a^Women who met all inclusion criteria for the women-only analyses.  ^b^Infants who met all inclusion criteria for the infant-only analyses.  ^c^Pairs who met all inclusion criteria for the maternal-infant pair analyses. | | | |

**Supplementary Table 2.** Geometric mean HAI titers and factor change between measurements by vaccine group.

| Vaccine component and treatment group  (n = women, infants) | **Mothers** | | | **Infants** | | |  |
| --- | --- | --- | --- | --- | --- | --- | --- |
|  | Baseline | One month | Factor change | Delivery | Six months | Factor change |  |
|  | *Geometric mean titer*  *(95% CI)* | | *Geometric mean ratio (95% CI)* | *Geometric mean titer*  *(95% CI)* | | *Geometric mean ratio (95% CI)* |  |
| *A/H1N1* |  |  |  |  |  |  |  |
| Controls^a^ | 24.1 | 25.5 | 1.1 | 17.7 | 6.9 | 0.4 |  |
| (n = 195, 117) | (20.0-29.2) | (21.0-31.1) | (1.0-1.2) | (13.8-22.5) | (6.0-8.1) | (0.3-0.5) |  |
| IIV3 recipients^b^ | 26.2 | **246.6** | **9.4** | **99.4** | **14.1** | **0.1** |  |
| (n = 209, 123) | (22.3-30.8) | (206.9-293.9) | (7.7-11.5) | (77.9-126.7) | (11.8-17.0) | (0.1-0.2) |  |
| *A/H3N2* |  |  |  |  |  |  |  |
| Controls^a^ | 20.7 | 23.5 | 1.1 | 17.9 | 8.2 | 0.5 |  |
| (n = 192, 114) | (17.2-24.9) | (19.3-28.5) | (1.0-1.2) | (14.3-22.5) | (6.9-9.7) | (0.4-0.6) |  |
| IIV3 recipients^b^ | 23.2 | **139.2** | **6.0** | **42.2** | 8.8 | 0.2 |  |
| (n = 195, 93) | (19.3-28.0) | (112.1-172.7) | (5.0-7.2) | (31.6-56.3) | (7.4-10.4) | (0.2-0.3) |  |
| *Type B* |  |  |  |  |  |  |  |
| Controls^a^ | 15.3 | 17.4 | 1.1 | 18.1 | 5.9 | 0.3 |  |
| (n = 192, 121) | (13.5-17.4) | (15.2-19.9) | (1.0-1.2) | (15.4-21.4) | (5.3-6.5) | (0.3-0.4) |  |
| IIV3 recipients^b^ | 16.4 | **158.1** | **9.6** | **71.1** | **8.2** | **0.1** |  |
| (n = 196, 97) | (14.7-18.3) | (132.5-188.7) | (8.1-11.5) | (56.6-89.4) | (7.1-9.3) | (0.1-0.1) |  |
| Two-sided, two-sample t-tests were used to compare the titers and factor change for each vaccine component between control and IIV3 recipient groups, with a log-normal distribution specified for the titers and factor change. Bold values indicate a p-value < 0.05.  ^a^Controls were women who received either meningococcal vaccine or a saline injection, or their infants.  ^b^IIV3 recipients were mothers who received IIV3, or their infants.  Abbreviations: HAI, hemagglutination-inhibition; CI: confidence interval; IIV3, Trivalent inactivated influenza vaccine | | | | | | | |

**Supplementary Table 3.** Proportion of women seroprotected (HAI ≥1:40) at each measurement and proportion who seroconverted (≥4x HAI titer increase between measurements).

| Vaccine component and treatment group (n = women) | Seroprotected at baseline | | Seroprotected at one month | | Seroconverted | | |
| --- | --- | --- | --- | --- | --- | --- | --- |
|  | *Proportion (95% CI)* | | | | | | |
| *A/H1N1* |  |  |  |  |  |  | |
| Controls^a^ (n = 195) | 39.5% | (32.6-46.7%) | 42.1% | (35.0-49.3%) | 6.7% | (3.6-11.1%) | |
| IIV3 recipients^b^ (n = 209) | 43.1% | (36.3-50.1%) | 93.3% | (89.0-96.3%) | **79.0%** | (72.8-84.3%) | |
| *A/H3N2* |  |  |  |  |  |  | |
| Controls^a^ (n = 192) | 32.8% | (26.2-39.9%) | 34.9% | (28.2-42.1%) | 3.7% | (1.5-7.4%) | |
| IIV3 recipients^b^ (n = 194) | 33.5% | (26.9-40.6%) | 83.0% | (77.0-88.0%) | **67.5%** | (60.5-74.1%) | |
| *Type B* |  |  |  |  |  |  | |
| Controls^a^ (n = 192) | **17.7%** | (12.6-23.9%) | **26.6%** | (20.5-33.4%) | 4.7% | (2.2-8.7%) | |
| IIV3 recipients^b^ (n = 196) | **16.3%** | (11.4-22.3%) | **90.8%** | (85.9-94.5%) | **86.7%** | (81.2-91.2%) | |
| Two-sided exact tests were used to compare the proportion seroprotected against each component within control and IIV3 recipient groups and the proportion who seroconverted between groups. Bold values indicate a p-value < 0.05.  ^a^Controls were women who received either meningococcal vaccine or a saline injection.  ^b^IIV3 recipients were women who received IIV3.  Abbreviation: HAI, hemagglutination-inhibition; CI: confidence interval; IIV3: trivalent inactivated influenza vaccine | | | | | | |  |

**Supplementary Table 4.** Geometric mean ratio of infant:mother HAI titers within one week of birth and proportion of infants seroprotected (HAI ≥1:40) at each measurement.

| Vaccine component and treatment group  (n = pairs, infants) | Transplacental transfer | | Seroprotected at delivery | | | Seroprotected at six months | | |
| --- | --- | --- | --- | --- | --- | --- | --- | --- |
|  | *Geometric mean ratio (95% CI)* | | *Proportion (95% CI)* | | | | | |
| *A/H1N1* |  |  |  |  |  | |  |  |
| Controls^a^ (n = 172, 117) | 0.8 | (0.7-0.9) | 32.5% | (24.1-41.8%) | 6.0% | | (2.4-11.9%) |  |
| IIV3 recipients^b^ (n = 197, 123) | 0.7 | (0.6-0.8) | 78.9% | (70.6-85.7%) | 20.3% | | (13.6-28.5%) |  |
| *A/H3N2* |  |  |  |  |  | |  |  |
| Controls^a^ (n = 168, 114) | 0.8 | (0.7-0.9) | 29.0% | (20.8-38.2%) | 7.9% | | (3.7-14.5%) |  |
| IIV3 recipients^b^ (n = 162, 93) | 0.6 | (0.6-0.8) | 58.1% | (47.4-68.2%) | 6.5% | | (2.4-13.5%) |  |
| *Type B* |  |  |  |  |  | |  |  |
| Controls^a^ (n = 171, 121) | 0.9 | (0.8-1.1) | 29.8% | (21.8-38.7%) | 1.7% | | (0.2-5.8%) |  |
| IIV3 recipients^b^ (n = 165, 97) | **0.8** | (0.7-0.9) | **77.3%** | (67.7-85.2%) | **8.3%** | | (3.6%-15.6%) |  |
| Two-sided, two-sample t-tests were used to compare transplacental transfer of HAI antibodies between control and IIV3 recipient groups, with a log-normal distribution specified for infant:mother ratio. Two-sided exact tests were used to compare the proportion seroprotected against each component within control and IIV3 recipient groups. Bold values indicate a p-value < 0.05.  ^a^Controls were mother-infants pairs where women received either meningococcal vaccine or a saline injection, or their infants.  ^b^IIV3 recipients were mother-infant pairs where women received IIV3, or their infants.  Abbreviations: HAI, hemagglutination-inhibition antibody; CI, confidence interval; IIV3, trivalent inactivated influenza vaccine; | | | | | | | | |

**Supplementary Table 5.** Proportion of women seroprotected (HAI ≥1:40) at each measurement and proportion who seroconverted (≥4x HAI titer increase between measurements), with women who tested positive for influenza prior to the one-month visit included.

| Vaccine component and treatment group (n = women) | Seroprotected at baseline | | Seroprotected at one month | | Seroconverted | |
| --- | --- | --- | --- | --- | --- | --- |
|  | *Proportion (95% CI)* | | | | | |
| *A/H1N1* |  |  |  |  |  |  |
| Controls^a^ (n = 195) | 39.5% | (32.6-46.7%) | 42.1% | (35.0-49.3%) | 6.7% | (3.6-11.1%) |
| IIV3 recipients^b^ (n = 209) | 43.1% | (36.3-50.1%) | 93.3% | (89.0-96.3%) | 79.0% | (72.8-84.3%) |
| *A/H3N2* |  |  |  |  |  |  |
| Controls^a^ (n = 194) | 32.5% | (25.9-39.6%) | 35.1% | (28.4-42.2%) | 4.6% | (2.1-8.6%) |
| IIV3 recipients^b^ (n = 196) | 33.7% | (27.1-40.8%) | 83.2% | (77.2-88.1%) | 67.9% | (60.8-74.3%) |
| *Type B* |  |  |  |  |  |  |
| Controls^a^ (n = 193) | 17.6% | (12.5-23.7%) | 26.4% | (20.4-33.2%) | 4.7% | (2.2-8.7%) |
| IIV3 recipients^b^ (n = 196) | 16.3% | (11.4-22.3%) | 90.8% | (85.9-94.5%) | 86.7% | (81.2-91.2%) |
| ^a^Controls were women who received either meningococcal vaccine or a saline injection.  ^b^IIV3 recipients were women who received IIV3.  Abbreviations: HAI, hemagglutination-inhibition; CI, confidence interval; IIV3, trivalent inactivated influenza vaccine | | | | | | |

**Supplementary Table 6.** Proportion of infants seroprotected (HAI titers ≥1:40) at birth, with infants of women who tested positive for influenza prior to their one-month visit included.

| Vaccine component and treatment group  (n = infants) | *Proportion (95% CI)* | | |
| --- | --- | --- | --- |
| *A/H1N1* |  |  | |
| Controls^a^ (n = 130) | 30.8% | (23.0-39.5%) | |
| IIV3 recipients^b^ (n = 130) | 78.5% | (70.4-85.2%) | |
| *A/H3N2* |  |  | |
| Controls^a^ (n = 131) | 29.0% | (21.4-37.6%) | |
| IIV3 recipients^b^ (n = 103) | 61.2% | (51.1-70.6%) | |
| *Type B* |  |  | |
| Controls^a^ (n = 131) | 29.0% | (21.4-37.6%) | |
| IIV3 recipients^b^ (n = 104) | 76.0% | (66.6-83.8%) | |
| ^a^Controls were infants of women who received either meningococcal vaccine or a saline injection.  ^b^IIV3 recipients were infants of women who received IIV3.  Abbreviations: HAI, hemagglutination-inhibition; CI, confidence interval; IIV3, trivalent inactivated influenza vaccine | | |  |

**Supplementary Table 7.** Proportion of women seroprotected (HAI ≥1:40) at each measurement and proportion who seroconverted (≥4x HAI titer increase between measurements). by vaccine strain.

| Vaccine component and treatment group (n = women) | Seroprotected at baseline | | Seroprotected at one month | | Seroconverted | | | | |  |
| --- | --- | --- | --- | --- | --- | --- | --- | --- | --- | --- |
|  | *Proportion (95% CI)^a^* | | | | | | |  |  |  |
| *A/H3N2 (Victoria/210/2009)* |  |  |  |  | |  |  | | | |
| Controls^a^ (n = 146) | 25.3% | (18.5-33.2%) | 26.7% | (19.7-34.7%) | | 2.7% | (0.8-6.9%) | | | |
| IIV3 recipients^b^ (n = 141) | 23.4% | (16.7-31.3%) | 78.7% | (71.0-85.2%) | | 68.8% | (60.5-76.3%) | | | |
| *A/H3N2 (Victoria/361/2011)* |  |  |  |  | |  |  | | | |
| Controls^a^ (n = 10) | 50.0% | (18.7-81.3%) | 50.0% | (18.7-81.3%) | | 0.0% | NA | | | |
| IIV3 recipients^b^ (n = 5) | 40.0% | (5.3-85.3%) | 100.0% | NA | | 80.0% | (28.4-99.5%) | | | |
| *A/H3N2 (Perth/16/2009)* |  |  |  |  | |  |  | | | |
| Controls^a^ (n = 36) | 58.3% | (40.8-74.5%) | 63.9% | (46.2-79.2%) | | 8.3% | (1.8-22.5%) | | | |
| IIV3 recipients^b^ (n = 48) | 62.5% | (47.4-76.1%) | 93.8% | (82.8-98.7%) | | 62.5% | (47.4-76.1%) | | | |
| *Type B (Brisbane/60/2008)* |  |  |  |  | |  |  | | | |
| Controls^a^ (n = 182) | 17.0% | (11.9-23.3%) | 26.4% | (20.1-33.4%) | | 5.0% | (2.3-9.2%) | | | |
| IIV3 recipients^b^ (n = 190) | 16.8% | (11.8-22.9%) | 90.5% | (85.4-94.3%) | | 86.8% | (81.2-91.3%) | | | |
| *Type B (Wisconsin/1/2010)* |  |  |  |  | |  |  | | | |
| Controls^a^ (n = 10) | 30.0% | (6.7-65.3%) | 30.0% | (6.7-65.3%) | | 0.0% | NA | | | |
| IIV3 recipients^b^ (n = 6) | 0.0% | NA | 100.0% | NA | | 83.3% | (35.9-99.6%) | | | |
| ^a^Controls were women who received either meningococcal vaccine or a saline injection.  ^b^IIV3 recipients were women who received IIV3.  Abbreviations: HAI, hemagglutination-inhibition; CI, confidence interval; IIV3, trivalent inactivated influenza vaccine | | | | | | | | |  |  |

**Supplementary Table 8.** Geometric mean ratio of infant:mother HAI titers within one week of birth and proportion of infants seroprotected (HAI titers ≥1:40) at or within one week of birth, by vaccine strain.

| Vaccine component and treatment group  (n = pairs, infants) | Transplacental transfer | | Seroprotected at delivery | |
| --- | --- | --- | --- | --- |
|  | *Geometric mean ratio (95% CI)* | | *Proportion (95% CI)* | |
| *A/H3N2 (Victoria/210/2009)* |  |  |  |  |
| Controls^a^ (n = 106, 83) | 0.7 | (0.6, 0.8) | 16.9% | (9.5-26.7%) |
| IIV3 recipients^b^ (n = 103, 81) | 0.7 | (0.6, 0.8) | 56.8% | (45.3-67.8%) |
| *A/H3N2 (Victoria/361/2011)* |  |  |  |  |
| Controls^a^ (n = 17, 12) | 1.0 | (0.8, 1.2) | 75.0% | (42.8-94.5%) |
| IIV3 recipients^b^ (n = 7, 4) | 0.7 | (0.3, 1.8) | 75.0% | (19.4-99.4%) |
| *A/H3N2 (Perth/16/2009)* |  |  |  |  |
| Controls^a^ (n = 45, 19) | 0.9 | (0.6, 1.2) | 52.6% | (28.9-75.6%) |
| IIV3 recipients^b^ (n = 52, 8) | 0.6 | (0.4, 0.9) | 62.5% | (24.5-91.5%) |
| *Type B (Brisbane/60/2008)* |  |  |  |  |
| Controls^a^ (n = 154, 107) | 0.9 | (0.8, 1.1) | 30.8% | (22.3-40.5%) |
| IIV3 recipients^b^ (n = 157, 89) | 0.8 | (0.7, 0.9) | 78.7% | (68.7-86.6%) |
| *Type B (Wisconsin/1/2010)* |  |  |  |  |
| Controls^a^ (n = 17, 14) | 0.8 | (0.6, 1.2) | 21.4% | (4.7-50.8%) |
| IIV3 recipients^b^ (n = 8, 8) | 0.5 | (0.2, 1.3) | 62.5% | (24.5-91.5%) |
| ^a^Controls were mother-infants pairs where women received either meningococcal vaccine or a saline injection, or their infants.  ^b^IIV3 recipients were mother-infant pairs where women received IIV3, or their infants.  Abbreviations: HAI, hemagglutination-inhibition; CI, confidence interval; IIV3, trivalent inactivated influenza vaccine | | | | |

**Supplementary Table 9.** Geometric mean HAI titers for women at one-month visit and factor change between baseline and one-month measurements by vaccine group and baseline immunological status. Women with baseline titers > 1:10 were considered to have pre-existing titers and immunologically-naïve otherwise.

| Vaccine component and treatment group  (n = naïve, pre-existing) | **Immunologically-Naïve Women** | | | **Women with Pre-Existing Titers** | | |  |
| --- | --- | --- | --- | --- | --- | --- | --- |
|  | One month | | Factor change | One month | | Factor change |  |
|  | *Geometric mean titer* | *(95% CI)* | *Geometric mean ratio (95% CI)* | *Geometric mean titer* | *(95% CI)* | *Geometric mean ratio (95% CI)* |  |
| *A/H1N1* |  |  |  |  |  |  |  |
| Controls^a^ (n = 73, 122) | 6.7 | (6.0-7.6) | 1.1 (1.0-1.2) | 56.7 | (46.3-69.4) | 1.0 (0.9-1.2) |  |
| IIV3 recipients^b^ (n = 52, 157) | 145.0 | (93.7-224.3) | 26.6 (17.2-40.9) | 294.0 | (246.0-351.5) | 6.7 (5.4-8.2) |  |
| *A/H3N2* |  |  |  |  |  |  |  |
| Controls^a^ (n = 86, 106) | 8.2 | (7.1-9.4) | 1.2 (1.1-1.4) | 55.2 | (44.0-69.1) | 1.1 (0.9-1.2) |  |
| IIV3 recipients^b^ (n = 76, 93) | 55.9 | (41.1-76.0) | 8.0 (5.9-10.8) | 253.4 | (198.6-323.3) | 5.0 (4.0-6.3) |  |
| *Type B* |  |  |  |  |  |  |  |
| Controls^a^ (n = 89, 103) | 9.0 | (7.7-10.4) | 1.2 (1.1-1.4) | 30.9 | (26.7-35.7) | 1.1 (0.9-1.2) |  |
| IIV3 recipients^b^ (n = 80, 116) | 100.2 | (74.7-134.4) | 13.0 (9.8-17.1) | 216.6 | (176.6-265.5) | 7.9 (6.3-9.8) |  |
| ^a^Controls were women who received either meningococcal vaccine or a saline injection.  ^b^IIV3 recipients were women who received IIV3.  Abbreviations: HAI, hemagglutination-inhibition; CI: confidence interval; IIV3, Trivalent inactivated influenza vaccine | | | | | | | |
